# Rapid identification of clinically interesting methanogens using an improved MALDI-TOF-MS assay

**DOI:** 10.1099/acmi.0.000372

**Published:** 2022-07-12

**Authors:** Cheick Oumar Guindo, Lynda Amir, Carine Couderc, Michel Drancourt, Ghiles Grine

**Affiliations:** ^1^​ IHU Méditerranée Infection, Marseille, France; ^2^​ Aix-Marseille-Université, IRD, IHU Méditerranée, MEPHI, Marseille, France; ^3^​ Laboratoire de Microbiologie, Assistance Publique à Marseille, IHU Méditerranée Infection, Marseille, France

**Keywords:** methanogens, culture, identification, multispacer sequence typing, MALDI-TOF-MS, clinical microbiology

## Abstract

Methanogens, the archaea uniquely detoxifying fermentative hydrogen into methane in the digestive tract, are increasingly being detected in pathology situations, rendering their rapid identification mandatory. We improved the experimental protocol to identify broth-cultured methanogens by matrix-assisted laser desorption time-of-flight MS (MALDI-TOF-MS). A database incorporating 34 reference spectra derived from 16 methanogen reference strains representative of eight species supported further identification of 21 *Methanobrevibacter smithii* and 14 *Methanobrevibacter oralis* isolates broth-cultured from human stool and oral fluid, respectively, with scores >2. In addition, MALDI-TOF-MS differentiated five *Methanobrevibacter smithii* genotypes incorporated in the study. The data reported here found MALDI-TOF-MS as a first-line identification method for methanogens recovered from microbiota and clinical samples.

## Introduction

Methanogens are acknowledged members of the digestive tract microbiota where these strictly aero-intolerant archaea detoxify molecular hydrogen issued from anaerobic bacterial fermentation into methane [1, 2]. They are the only sources of methane [2]. Accordingly, the oral and gut cavities of virtually all humans harbour methanogens, with respect to this indispensable physiological role, and eight different species of methanogens have been detected and cultured from the oral and intestinal microbiota [3, 4]. More specifically, *Methanobrevibacter smithii *, *Methanobrevibacter oralis* and *Methanobrevibacter massiliense* have been isolated from the oral cavity, whereas *Methanobrevibacter smithii, Methanobrevibacter oralis*, *Methanosphaera stadtmanae, Methanomassiliicoccus luminyensis*, *Methanobrevibacter arboriphilicus,* ‘*Candidatus*’ Methanomethylophilus alvus and ‘*Candidatus*’ Methanomassiliicoccus intestinalis have been isolated from stools [3, 5, 6]. Other methanogens including *Methanosarcina mazei *, *Methanoculleus chikugoensis *, *Methanoculleus bourgensis* and *Methanobacterium congolense* have been PCR-detected in the gut microbiota but not cultured yet from clinical specimens [4].

Methanogens are emerging pathogens increasingly detected in various situations of pathology, such as dysbiosis, abscesses [2] and more recently archaeamia [6]. In all these pathological situations, methanogens have always been detected in the presence of bacteria, including *Enterobacteriaceae *, *Staphylococci* and *Streptococci*.

Owing to this increasing role in diverse pathological processes supported by the isolation and culture of methanogens in the clinical microbiology laboratory, there is a demand for a routine and specific method of identification of these unusual microorganisms in clinical microbiology. Variable microscopic morphological aspects ranging from single coccobacillary for *Methanobrevibacter smithii *, pairs or short chains oval rods for *Methanobrevibacter oralis *, single cell cocci for *Methanomassiliicoccus luminyensis* and pairs or tetrads cocci for *Methanosphaera stadtmanae *, may not be specific enough for the accurate identification of methanogens [5]. Fluorescence *in situ* hybridization (FISH) has added to specificity of the microscopic examination of methanogens in clinical specimens but remains too laborious for a routine usage [7]. Accordingly, the firm identification of cultured methanogens still relies upon the detection of species–species DNA sequences [7, 8]. However, DNA-based identification methods delay identification as current archaeal DNA extraction protocols add 3–24 h to the 1 h real-time PCR protocol or 3 h PCR-sequencing protocol when looking for methanogen species [9]. Potential carry-over of DNA resulting in false-positive results as well as the cost of PCR-based protocols may also limit their use for the routine identification of methanogen colonies, so a more appropriate method is warranted.

Matrix-assisted laser desorption ionization time-of-flight MS (MALDI-TOF-MS) is currently the first-line method for the identification of bacteria and yeasts cultured in clinical microbiology as this method precisely overcomes the potential limitations of PCR-based methods as reported above [10]. In addition, the benefit of MALDI-TOF over sequencing is that the downstream process is quicker, which is important from a screening/clinical point of view [10]. The rapid and reproducible identification of some environmental archaea by MALDI- TOF-MS has been reported by some authors [11, 12]. In addition, in another study, the authors succeeded in identifying by MALDI- TOF-MS some strains of methanogens of clinical interest for humans at a time when isolation and culture of methanogens was still in its infancy in clinical microbiology laboratories [13]. However, the authors of that study used a slow protein extraction protocol including the use of glass beads during the lysis phase [13], making it very difficult to use this method for routine identification of methanogens of clinical interest to clinical microbiology laboratories.

Here, following improvements in experimental and informatic protocols, we are reporting on the MALDI-TOF-MS identification of broth-cultured methanogens, renewing interest in MALDI-TOF-MS-based identification of methanogens of clinical interest.

## Methods

### Methanogen clinical isolates and strains

Sixteen reference strains of methanogens available in the Collection de Souches de l’Unité des Rickettsies (CSUR, Marseille, France) were used to create a MALDI-TOF-MS reference database (Table 1). These 16 reference strains of methanogens were subcultured in broth using a culture protocol previously established in our laboratory [14] and their identification was firmly confirmed by PCR sequencing of the 16S rRNA gene, as previously described [15]. Further clinical isolates were made from mucosa-associated specimens as previously described [16, 17]. In total, 35 clinical isolates included 21 stool isolates and 14 oral swab isolates, identified by PCR sequencing targeting 16S rRNA sequences of Archaea and cultured in broth for 9 days before being assessed by MALDI-TOF-MS against the MALDI-TOF reference database. Furthermore, we performed multispacer sequence typing (MST) of 21 *Methanobrevibacter smithii* clinical isolates. The MST technique and analysis was performed on each of the *Methanobrevibacter smithii* clinical isolates according to a previously described protocol [9, 18–20]. This part of the study was previously approved by the Ethics Committee of the University-Hospital Institute (IHU) Méditerranée Infection under no. 2016-020 and participants were given all information about the process of the study and gave informed consent before participation in this study.

**Table 1.** Methanogen reference strains used in this study

Species	Genotype	Source	CSUR number	Origin
* Methanomassiliicoccus luminyensis *	na	IHU [21]	CSURP9636	Human stool
* Methanosphaera stadtmanae *	na	DSMZ	CSURP9634	Human stool
* Methanobrevibacter arboriphilicus *	na	DSMZ	CSURP9635	Human stool
* Methanobrevibacter smithii *	Genotype 3	IHU [16, 17]	CSURP5816	Human stool
* Methanobrevibacter smithii *	Genotype 2	IHU [16, 17]	CSURP5922	Human stool
* Methanobrevibacter smithii *	Genotype 5	IHU [16, 17]	CSURQ5493	Human stool
* Methanobrevibacter smithii *	Genotype 1	IHU [16, 17]	CSURQ5497	Human stool
* Methanobrevibacter smithii *	Genotype 6	IHU [16, 17]	CSURQ5501	Human stool
* Methanobrevibacter oralis *	na	IHU [16, 17]	CSURP5701	Oral fluid
* Methanobrevibacter oralis *	na	IHU [16, 17]	CSURQ5479	Oral fluid
* Methanobrevibacter oralis *	na	IHU [16, 17]	CSURQ5481	Oral fluid
* Methanobrevibacter oralis *	na	IHU [16, 17]	CSURQ5483	Oral fluid
* Methanobrevibacter oralis *	na	IHU [16, 17]	CSURQ5485	Oral fluid
* Methanosarcina mazei *	na	DSMZ	CSURP9637	Environment
* Methanosarcina barkeri *	na	DSMZ	CSURP9601	Environment
* Methanobacterium beijingense *	na	DSMZ	CSURP9638	Environment

DSMZ, German Collection of Microorganisms and Cell Cultures; IHU, University-Hospital Institute; na, Not applicable;

### Methanogen MALDI-TOF-MS reference database

We used a protocol provided by the manufacturer MALDI Biotyper manufacturer (Bruker Daltonics), adding two additional washing steps to remove the culture medium as well as extending the centrifugation time after the ethanol addition step. Briefly, 1 ml of archaeal suspension corresponding to 108 UFC ml^–1^ was transferred to a sterile Eppendorf tube (Fisher Scientific) and centrifuged at 17 000 ***g*** for 30 min. The pellet was suspended in 500 µl High Purity Liquid Chromatography water or HPLC water (VWR International), vortexed and centrifuged at 17 000 ***g*** for 10 min, and this washing step was repeated twice. The pellet was suspended in 300 µl HPLC water (VWR International), homogenized by pipetting, and 900 µl of ethanol absolute for HPLC Chromanorm (VWR International) was added and homogenized by pipetting. After 5 min of centrifugation at 17 000 ***g***, the pellet was suspended in 50 µl of 70 % formic acid and 50 µl acetonitrile and vortexed for 10 s. After a final 2 min of centrifugation at 17000 ***g***, 1.5 µl of supernatant was deposited in a MALDI-TOF 96 MSP target polished steel BC ref number: 8280800 (Bruker Daltonik) and 10 spots were deposited for each methanogen species. After drying, each spot was coated with 1.5 µl of a matrix solution consisting of saturated α-cyano-4-hydroxycynnamic acid or HCCA (C2020; Sigma,), 50 % acetonitrile (CarboErba for HPLC) 2.5 % trifluoroacetic acid or TFA (Uvasol for spectrometry; Sigma-Aldrich) and 47.5 % HPLC water (VWR International). After drying in ambient air, the target plate was introduced into the Microflex LT MALDI-TOF-MS device (Bruker Daltonics). Each spot was then analysed with the help of FlexControl version 3.4 acquisition software and MALDI Biotyper Compass version 4.1.80 (MBT Compass) analysis software. The positive control consisted of a protein extract Bacterial Test Standard Bruker ref: 8255343. Non-inoculated media and matrix solutions were used as negative controls. Also, to prevent any cross-contamination, every MALDI-TOF plate (Bruker Daltonics) used in the study was thoroughly cleaned to remove any previous deposits of other microorganisms. The MALDI-TOF plate was first soaked in 70 % ethanol (Absolute for HPLC Chromanorm) for 15 min and then rinsed with HPLC water (VWR International). We subsequently stripped the MALDI-TOF plate with 500 µl of 80 % TFA (Sigma-Aldrich) while gently scrubbing and then rinsed with HPLC water (VWR International), to thoroughly clean the plate under a chemical hood.

### Reference MALDI-TOF profiling reproducibility

For each of the 16 methanogen reference strains tested, 10 deposits were made on the MALDI-TOF-MS plate and all experiments were performed on day 9 of culture, corresponding to the optimal growth period based on a Ct score of 18 using real-time PCR [14, 17, 19].

### Blind MALDI-TOF identification and clustering of methanogen clinical isolates

MALDI Biotyper version 4.1.80 software (Bruker Daltonics) was used to create reference spectra for blind identification. Spectral references (MSP) were created using the Biotyper MSP Creation Standard Method. Five raw spectra were used to create an MSP for each species. We performed the experiments in triplicate and a total of 34 MSPs were found to be of very good quality and therefore used as a reference database. We compared the 35 clinical isolates to this database.

## Results and discussion

We improved the MALDI-TOF-MS protocol for identifying cultured methanogens after a specific database was created, allowing for the rapid and routine identification of colonies originating from clinical specimens collected from mucosa-associated specimens and pathological specimens. The data reported here were validated based the negativity of the negative controls introduced in each of the experimental steps and the reproducibility of the data, which were correlated with PCR sequencing molecular identification used as the gold standard.

**Fig. 1.**
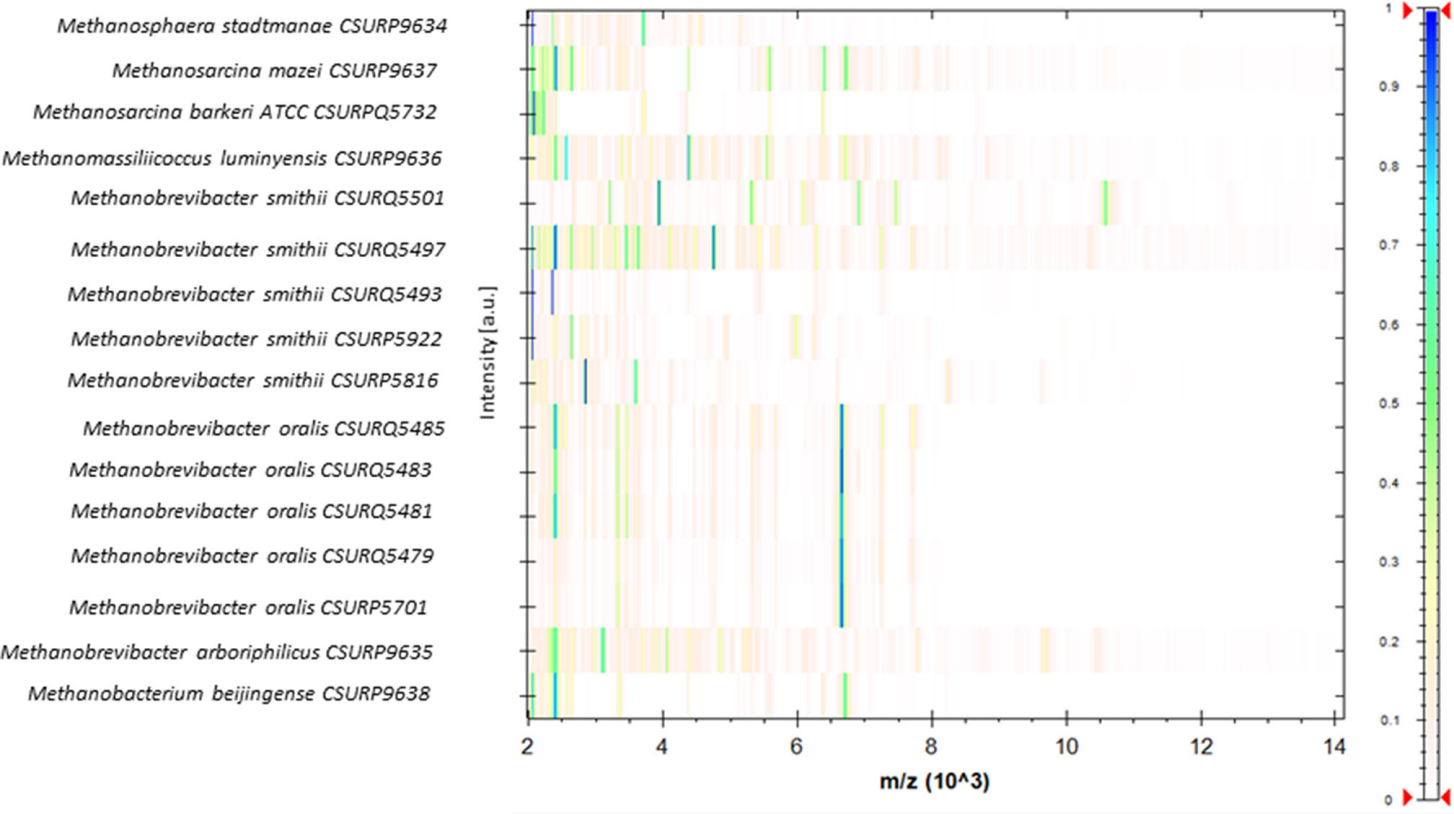
Comparison of the MALDI-TOF-MS spectra of the 16 reference methanogen strains using MALDI Biotyper version 4.1.80 software (Bruker Daltonics). Normalized gel view representation of the MS profile intensity of the 16 reference methanogenic strains used in this study. **a.**u., Arbitrary units; m/z, mass-to-charge ratio.

**Fig. 2.**
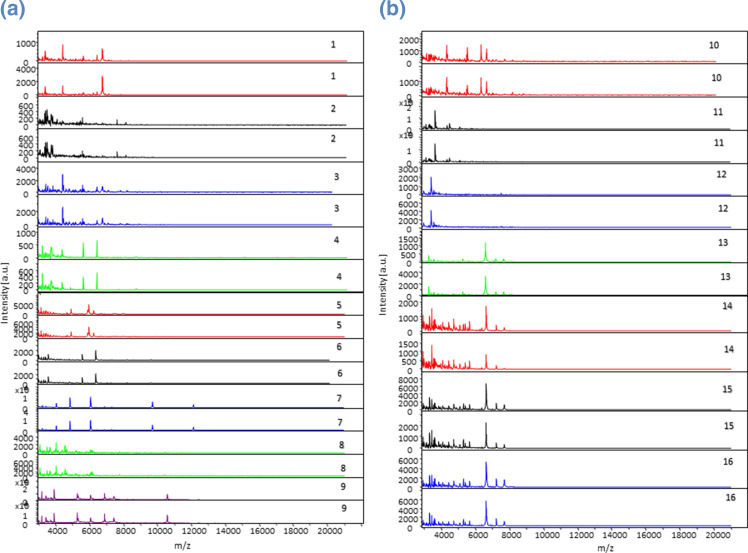
Representative MS profiles of the 16 methanogen reference strains studied using FlexAnalysis software. (1) MS profiles of *Methanobacterium beijingense* CSURP9638. (2) MS profiles of *Methanobrevibacter arboriphilicus* CSURP9635. (3) MS profiles of *M. luminyensis* CSURP9636. (4) MS profiles of *Methanosarcina barkeri* CSURQ5732. (5) MS profiles of *Methanobrevibacter smithii* CSURP5922. (6) MS profiles of *Methanobrevibacter smithii* CSURP5816. (7) MS profiles of *Methanobrevibacter smithii* CSURQ5493. (8) MS profiles of *Methanobrevibacter smithii* CSURQ5497. (9) MS profiles of *Methanobrevibacter smithii* CSURQ5501. (10) MS profiles of *Methanosarcina mazei* CSURP9637. (11) MS profiles of *Methanosphaera stadtmanae* CSURP9634. (12) MS profiles of *Methanobrevibacter oralis* CSURP5701. (13) MS profiles of *Methanobrevibacter oralis* CSURQ5479. (14) MS profiles of *Methanobrevibacter oralis* CSURQ5481. (15) MS profiles of *Methanobrevibacter oralis* CSURQ5483. (16) MS profiles of *Methanobrevibacter oralis* CSURQ5485. The 16 methanogen reference strains are presented in duplicate to demonstrate the reproducibility of MS profiles. a.u., Arbitrary units; m/z, mass-to-charge ratio.

**Fig. 3.**
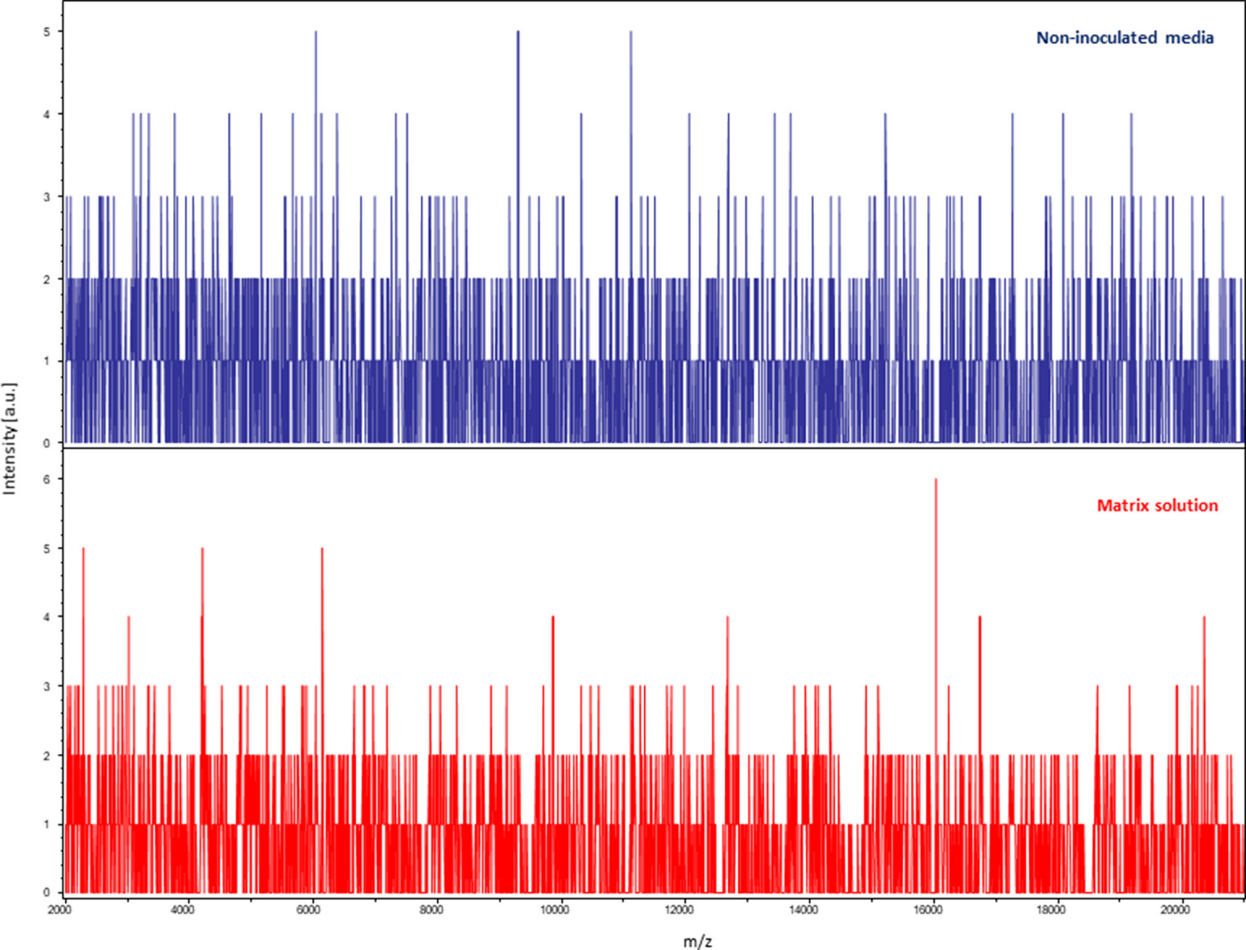
Negative controls used in the MALDI-TOF-MS experiment. Non-inoculated media are in blue and matrix solution in red. **a.**u., Arbitrary units; m/z, mass-to-charge ratio.

The database was created after three independent runs, each incorporating 10 spots for each one of 16 methanogen reference strains. We obtained a total of 270 spectra and 135 spectra (50%) of good quality for analysis, which were incorporated in the database. We therefore set up a database with 34 reference spectra. We then blindly tested the identification of these 16 reference strains using the updated local database by analysing five spots for each of them. We obtained a correct identification for these different spots with scores >2 for each of the reference strains. We observed a unique protein profile for each of the methanogen reference strains studied ((Figs. 1 and 2). We did not observe any spectra on the negative controls (Fig. 3). There are 15 different MST genotypes of *Methanobrevibacter smithii* among *Methanobrevibacter smithii* strains known in humans according to a previous study [18]. MST is used as the first-line method for genotyping *Methanobrevibacter smithii* strains found in humans. It allows the differentiation of *Methanobrevibacter smithii* strains isolated from humans [18]. We observed a difference in the number and intensity of spectra between *Methanobrevibacter smithii* strains from different MST genotypes (Fig. 2b). These observations showed the reproducibility and the inter-species specificity of the method. These data confirmed the ability of MALDI-TOF-MS to identify archaea cultured in broth, as previously reported in a proof-of-concept study using a difficult protein extraction protocol [13]. We further tested our database against all the MALDI-TOF-MS databases available in our laboratory, incorporating 16 908 spectra representative of 12 290 different microbial species (i.e. 4335 bacteria, 5989 parasites and 1966 fungi), and no identification was observed, revealing the specificity of our spectra and an absence of contaminating spectra. Genotyping the 21 clinical isolates of *Methanobrevibacter smithii* revealed five different genotypes. Genotype 1 was found in 10/21 (47.76 %), genotype 2 in 5/21 (23.80 %), genotype 3 in 3/21 (14.28 %), genotype 5 in 2/21 (9.52 %) and genotype 6 in 1/21 (4.76 %). These results confirm the predominance of the MST1 genotype in clinical isolates of *Methanobrevibacter smithii* found in the human digestive tract [9, 19].

We then used the reference database for the identification of clinical isolates of *Methanobrevibacter smithii* and *Methanobrevibacter oralis* obtained by broth culture of human stool and oral fluid, respectively. We obtained a correct identification of all the clinical isolates tested with scores >2, i.e. 21/21 (100 %) for *Methanobrevibacter smithii* and 14/14 (100 %) for *Methanobrevibacter oralis,* respectively. We noted that for good identification by MALDI-TOF-MS of *Methanobrevibacter smithii *, it is necessary to incorporate a larger set of reference spectra of *Methanobrevibacter smithii* of different MST genotype in the reference database due to the diversity observed within this species [9, 18, 19], which results in different spectral profiles depending on each MST genotype as obtained in our study. To confirm this hypothesis, we repeated the blind test with only two reference strains of *Methanobrevibacter smithii* with different MST genotype, i.e. *Methanobrevibacter smithii* CSURP5816 of MST genotype 3, and *Methanobrevibacter smithii* CSURP5922 of MST genotype 2. We observed that with these two reference strains, we obtained only 23 % (5/21) correct identification with scores >2 and 19 % (4/21) with scores >1.7. Our results demonstrate the need for rapid expansion of the MALDI-TOF-MS database to incorporate isolates of clinical interest.

The results here confirmed the proof-of-concept study carried out in our laboratory in which MALDI-TOF-MS was able to correctly identify the methanogens and environmental archaea [13]. We further observed that MALDI-TOF-MS could differentiate between different genotypes of *Methanobrevibacter smithii *, opening the possibility of using MALDI-TOF-MS as a first-line typing method for this species of prime clinical interest. We are implanting the experimental protocol and MALDI-TOF-MS methanogen database in the workflow of our clinical microbiology laboratory for the routine, rapid identification of methanogens of clinical interest. The MALDI-TOF MS database created in this study is available on the website of the University-Hospital Institute (IHU) Méditerranée Infection via the link https://www.mediterranee-infection.com/acces-ressources/base-de-donnees/urms-data-base/. This database is accessible to the entire scientific community.
